# The Impact of Polyethylene Glycol-Modified Chitosan Scaffolds on the Proliferation and Differentiation of Osteoblasts

**DOI:** 10.1155/2023/4864492

**Published:** 2023-01-03

**Authors:** Wei-Bor Tsai, Ibrahim Nasser Ahmed

**Affiliations:** ^1^Department of Chemical Engineering, National Taiwan University, No. 1 Sec. 4 Roosevelt Rd., Taipei 106, Taiwan; ^2^Nanotechnology Center of Excellence, Addis Ababa Science and Technology University, P.O. Box 16417, Addis Ababa, Ethiopia; ^3^Department of Industrial Chemistry, College of Applied Sciences, Addis Ababa Science and Technology University, P.O. Box 16417, Addis Ababa, Ethiopia

## Abstract

The objective of this study was to investigate the influence of polyethylene glycol (PEG) incorporated chitosan scaffolds on osteoblasts proliferation and differentiation. The chitosan polymer was initially modified by the predetermined concentration of the photoreactive azido group for UV-crosslinking and with RGD peptides (N-acetyl-GRGDSPGYG-amide). The PEG was mixed at different ratios (0, 10, and 20 wt%) with modified chitosan in 96-well tissue culture polystyrene plates to prepare CHI-100, CHI-90, and CHI-80 scaffolds. PEG-containing scaffolds exhibited bigger pore size and higher water content compared to unmodified chitosan scaffolds. After 10 days of incubation, the cell number of CHI-90 (1.1 × 106 cells/scaffold) surpasses that of CHI-100 (9.2 × 105 cells/scaffold) and the cell number of CHI-80 (7.6 × 105 cells/scaffold) were significantly lower. The ALP activity of CHI-90 was the highest on the fifth day indicating the favored osteoblasts' early-stage differentiation. Moreover, after 14 days of osteogenic culture, calcium deposition in the CHI-90 scaffolds (2.7 *μ*mol Ca/scaffold) was significantly higher than the control (2.2 *μ*mol Ca/scaffold) whereas on CHI-80 was 1.9 *μ*mol/scaffold. The results demonstrate that PEG-incorporated chitosan scaffolds favored osteoblasts proliferation and differentiation; however, mixing relatively excess PEG (≥20% wt.) had a negative impact on osteoblasts proliferation and differentiation.

## 1. Introduction

Chitosan, the deacetylated derivative of chitin, has been broadly utilized for the fabrication of tissue engineering scaffolds due to its nontoxicity, biodegradability, good biocompatibility [1], and resemblance to glycosaminoglycans [2]. However, chitosan still possesses some shortcomings for such a purpose, for instance, the mechanical properties of chitosan scaffolds may not be suitable to match some specific tissue engineering applications. Previously it was utilized an azide-based UV-crosslinking mechanism for crosslinking chitosan scaffolds in order to increase the mechanical properties of chitosan scaffolds [3–5]. Upon UV irradiation, the azido groups are converted into highly reactive nitrene groups which undergo direct insertion into C–H, O–H, and N–H bonds of nearby substance molecules [6]. Another shortcoming is that chitosan lacks bioactive signals equivalent to those existing in the extracellular matrix (ECM) for cell attachment, growth, and differentiation. Incorporation of bioactive signals such as ECM adhesion proteins and cell-binding peptides into chitosan substrates can enhance cell adhesion [7–9]. RGD-incorporated and crosslinked chitosan scaffolds can be employed for mesenchymal stem cell proliferation and osteogenic differentiation [3, 4].

There are many reports aimed at improving chitosan properties by blending with natural or synthetic molecules. Park et al. [10] developed composite chitosan scaffolds containing anionic carbohydrates. The incorporation of chondroitin 4-sulfate or alginate in chitosan scaffolds increased the compressive modulus of the scaffolds and enhanced apatite formation. Furthermore, apatite-coated scaffolds enhanced the spreading, proliferation, and osteogenic differentiation of bone marrow stromal cells seeded on the scaffolds. A report by Li et al. [11] using a 3-aminopropyltriethoxysilane treatment for modification and biocompatibility of lyophilized chitosan porous scaffolds showed the silanization treatment with low 3-aminopropyltriethoxy silane concentration showed no significant influence on the morphology of chitosan scaffolds, the porosity, and surface amino densities were increased after silanization whereas the swelling ratio was reduced, and the degradation ratio in PBS and antiacid degradation properties of the silanized chitosan scaffolds were significantly improved. Chitosan doped with multiwalled carbon nanotubes has been used to create highly porous conductive scaffolds [12]. Chitosan can also be modified by the addition of hydrophobic alkyl chains along the hydrophilic backbone of the chitosan polymer and Cooney et al. [13] reported the scaffolds produced from the unmodified chitosan were more stable and rigid and possessed average pore diameters that were generally smaller than those fabricated from the hydrophobically modified chitosan. The generally larger pores in the butyl-modified chitosan scaffolds might be explained by increased phase separation rates due to the introduced hydrophobicity of the chitosan polymer. The combination of hydrophobic groups opposed along an otherwise hydrophilic backbone creates internal forces that tend to fold or buckle the polymer chain, creating regions that exhibit micellar behavior [14].

One major drawback of chitosan in drug delivery is its low solubility. Chitosan is not soluble in aqueous solutions at neutral or alkaline pH, only soluble in aqueous acid solutions and a few organic solvents [15]. Hence, various chitosan derivatives have been prepared for the purpose of drug delivery [15, 16]. Similarly, in this work it was argued that chitosan is a relatively hydrophobic material; however, in the natural tissues, the extracellular matrix is highly hydrated. Thus, the hydrophobic environment of chitosan scaffolds might not be suitable for tissue growth. A number of studies indicate that both morphology and hydrophilicity influence the attachment of cells onto the surface of a scaffold [17]. Therefore, an increase in the hydrophilicity of chitosan scaffolds might improve tissue engineering outcomes. Polyethylene glycol (PEG) possesses biodegradability, biocompatibility, less toxicity, and hydrophilicity and has been widely used in biomedical applications, including surface modification, bioconjugation, drug delivery, and tissue engineering [18, 19]. Although there are interesting contributions to the preparation, characterization, and aggregation behavior of amphiphilic chitosan derivatives having poly-L-lactic acid side chains [20], there is no report on describing the osteogenic proliferation or differentiation of cells, from hydrophilically modified chitosan scaffold. Hence, this study aimed to investigate the effect of adding polyethylene glycol into the RGD-conjugatedcross-linked chitosan scaffolds on the proliferation and differentiation of osteoblasts.

## 2. Material and Methods

### 2.1. Material

Polyethylene glycol *M*_*n*_ 20,000 and most of the reagents were purchased from Sigma–Aldrich (USA) unless specified otherwise. N-(3-dimethylaminopropyl)-N′-ethylcarbodiimide hydrochloride was purchased from Fluka (USA), and acetic acid was purchased from Baker (USA). RGD-peptide (N-acetyl-GRGDSPGYG-amide) was synthesized by Kelowna International Scientific Inc. (Taipei, Taiwan). The peptide concentration was calculated by absorbance at 275 nm coming from the tyrosine residue (Y, molar adsorption coefficient 1420 M^−1^ cm^−1^).

Osteoblast culture medium contained *α*-minimum essential medium (*α*-MEM, HyClone, USA), 10% fetal bovine serum (JRH Biosciences, Australia), 0.0679% (v/v) 2-mercaptoethanol, 200 *μ*g/mL gentamicin (GIBCO®, Invitrogen, USA), and 25 *μ*g/mL fungizone (GIBCO®), pH 7.4. The osteoblast culture medium supplemented with 1 mM sodium glycerophosphate, 0.1 *μ*M dexamethasone, and 50 *μ*g/mL L-ascorbate constituted osteoblast differentiation medium. Phosphate buffered saline (PBS) contained 137 mM NaCl, 2.7 mM KCl, 10 mM Na_2_HPO_4_, and 1.8 mM KH_2_PO_4_ (pH 7.4).

### 2.2. Conjugation of Photoreactive Azido Groups or Peptides to Chitosan

Azido groups were conjugated onto chitosan (molecular weight 50–190 kDa, 75–85% deacetylation) via a reaction forming covalent amide bonds between the amino groups of chitosan and the carboxyl groups of an azidobenzoic acid ester. Briefly, 17.6 mg 5-azido-2-nitrobenzoic acid N-hydroxysuccinimide ester was dissolved in 200 *μ*L dimethylsulfoxide and then mixed with chitosan solution (0.1 g in 4.8 mL of 1% acetic acid), followed by 3 h incubation at room temperature. The unreacted azido ester was removed by dialysis against deionized water through a seamless cellulose tube (MWCO 12,400 Da) in the dark for two days with the changes of deionized water every 12 h. After freeze-drying, the azido-conjugated chitosan (CHI-g-AZ) was kept at 4°C until use.

RGD peptides were conjugated onto chitosan molecules via a carbodiimide reaction according to a previously developed procedure [5]. The graft ratio of RGD to chitosan (CHI-g-RGD) was estimated as 2.75 mol% with respect to the total moles of the amino groups of chitosan molecules.

### 2.3. Preparation of Chitosan and polyethylene Glycol Mixed Scaffold

A mixture of chitosan (unmodified, CHI-g-AZ, and CHI-g-RGD) and PEG with a total concentration of 10 mg/mL in 1% acetic acid was prepared at different weight percentages (the composition and abbreviations listed in Table 1). Chitosan-PEG mixed scaffolds were prepared by adding 70 *μ*L/well of the unmodified chitosan, CHI-g-AZ, CHI-g-RGD, and PEG mixture in 96-well tissue culture polystyrene (TCPS) plates. Briefly, the mixture was poured into 96-well TCPS plates (70 *μ*L/well for cell culture experiments), followed by freeze-drying in the dark to form scaffolds. Subsequently, chitosan substrates were crosslinked by UV irradiation for 30 min (wavelength range 280–380 nm). The UV crosslinking time and the PEG dose were selected based on the previous reports [5, 21].

### 2.4. Characterization of Chitosan Scaffolds

The morphology of chitosan-PEG scaffolds was observed by scanning electron microscope (SEM) images (JSM-5310, JEOL, Japan). The scaffolds were first dehydrated in graded series of ethanol solutions 30%, 50%, 70%, 90%, 95%, and 100% for 10 min each step followed by CO_2_ critical point-drying. Samples were cut with a scalpel, coated with a gold layer on the section, and then observed with SEM at an acceleration voltage of 20 kV.

The pore sizes of the scaffolds were analyzed using an NIH Image J. Pores in SEM images were traced manually, and the enclosed areas and perimeters of pores were determined by the NIH Image J software. The hydraulic diameters of the pores were determined by the following equation: pore diameter (*D*_*p*_) = 4 × area/perimeter [5] more than 100 pores were counted for each type of sample.

The compressive stress-strain properties of the scaffolds were determined using a compressive testing machine (FGS-50V-H, NIDECSIMPO Corporation, Japan) and a digital force gauge (FGP-0.5, NIDECSIMPO Corporation, Japan). The scaffolds were subjected to an unconfined uniaxial compression to 70% strain at a compression velocity of 3 mm/s. The continuous stress and peak stress were recorded and analyzed.

Dried chitosan-PEG scaffolds were soaked in deionized water for 24 h. The surface water contents on the scaffolds were absorbed by a filter paper. Wet scaffolds were weighed (*W*_*s*_), and then placed in a 70°C oven overnight and weighed again (*W*_*d*_). The equation implemented to calculate water content is shown as follows:

(1) $$\begin{array}{c} Water\;content\;percentage\left(\%\right)=\frac{\left(Ws-Wd\right)}{Wd}\left(\%\right)\times 100\%\text{.} \end{array}$$

### 2.5. Culture of Osteoblasts on Chitosan Scaffolds

Standard sterile cell culture techniques were used for all cell experiments. The animal procedure was followed by the ethical guidelines of Care and Use of Laboratory Animals (National Taiwan University, National Institutes of Health Publication No. 85–23, revised 1985) and was approved by the Animal Center Committee of National Taiwan University. Primary osteoblasts were isolated from neonatal rat calvariae according to the previously published procedure [22]. The number and viability of the isolated osteoblasts were determined using a hemocytometer with trypan blue exclusion. The isolated cells were cultured in standard T75 flasks to the second passage for the cell experiments.

Prior to cell seeding, the chitosan-PEG scaffolds were soaked in 70% ethanol for 30 min, followed by rinses with sterilized PBS three times. For cell culture on the scaffolds, 20 *μ*L of osteoblast suspension (1.5 × 10^7^ cells/mL) was seeded onto scaffolds, making the seeding density 3 × 10^5^ cells per scaffold. After 1, 5, or 10 days of culture, the cell-inoculated samples were analyzed for cell morphology cell numbers and alkaline phosphatase (ALP) activities.

After the cell culture, the morphology of cells in the scaffolds after 5 days of incubation was observed by SEM. The adhered osteoblasts were lysed with 0.1% Triton X-100 for 30 min. Cell proliferation was determined by the lactate dehydrogenase (LDH) method according to a reported protocol [9]. Intracellular alkaline phosphatase activities were assayed by determining the release of p-nitrophenol from 4-nitrophenyl phosphate disodium salt at pH 10.2, as reported previously [23].

The cell doubling time (*T*_2_) was calculated using the following equation:

(2) $$\begin{array}{c} T_{2}=\frac{∆T}{\log_{2}\;\left(∆N/No+1\right)}\text{.} \end{array}$$

No is the number of cells at the beginning of the observation, and △*N* is the increase in the number of cells during the period of time of the length △*t*. Each division increases the number of cells by adding 1, and △*N* is also the number of cell divisions during the same period [24].

### 2.6. Mineralization Culture of Osteoblast/Scaffold Constructs

Osteoblast/scaffold constructs were cultured for 5 days in the osteoblast culture medium, followed by 10 days of mineralization culture in the osteoblast differentiation medium with daily replenishment of L-ascorbate (50 *μ*g/mL). The total amount of calcium deposition was determined using a calcium assay kit (Diagnostic Chemicals Limited, USA) [25].

### 2.7. Statistical Analysis

Each experiment has been repeated at least three times. The data were presented as mean ± standard deviation (SD). The statistical assessment of significant variations was performed by Microsoft Excel 2010. Significance was assessed by one-way analysis of variance (ANOVA) and two-tailed Student–Newman–Keuls multiple comparisons. The probability of *p* ≤ 0.05 was considered as a significant difference, where the symbol of ∗ and ∗∗ marker represent *p* < 0.05 and *p* < 0.01, which is of significant difference statistically in 95% and 99% confidence level, respectively.

## 3. Results and Discussion

### 3.1. Fabrication and Characterization of Chitosan/PEG Scaffolds

**Figure 1:**
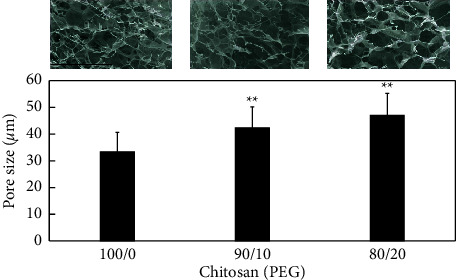
SEM micrographs and the corresponding pore size of CHI-100 (100/0), CHI-90, and CHI-80 (80/20) scaffolds. The scale bar in the images represents 300 *μ*m. The values represent mean ± standard deviation, *n* = 4. ^∗^indicates *p* < 0.05 vs. PEG-100 and PEG-90 or PEG-100 and PEG-80.

Chitosan has a structure alike the N-acetylglucosamine, which exists in hyaluronic acid, is an extracellular macromolecule, and it is vital in wound healing [26]. The morphology of the chitosan scaffolds incorporating PEG was examined with SEM. All the scaffolds exhibited an open pore microstructure with interconnectivity. The pore structure of the scaffolds at different PEG concentrations is similar to each other (Figure 1). The average pore sizes of CHI-100, CHI-90, and CHI-80 were 33.3 ± 7.4, 42.2 ± 8.2, and 46.9 ± 8.6 *μ*m, respectively, indicating the pore sizes of the scaffolds were significantly increased (*p* < 0.05) with the increasing PEG contents in chitosan scaffolds (Figure 1). It was suspected that since PEG is more hydrophilic than chitosan, more water molecules surround PEG and form larger ice crystals during the freezing step than pure chitosan scaffolds. As a result, after lyophilization chitosan/PEG scaffolds contain larger pores compared with pure chitosan scaffolds.

**Figure 2:**
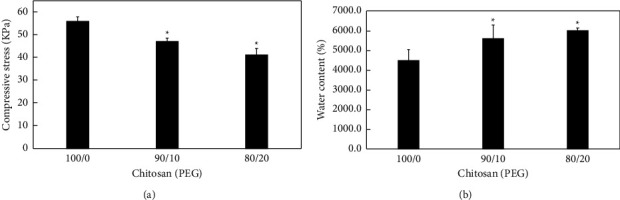
(a) Compressive modulus of CHI-100, CHI-90, and CHI-80 scaffolds. The values represent mean ± standard deviation, *n* = 4. ^∗^indicates *p* < 0.05 vs. PEG-100 and PEG-90; PEG-100, and PEG-80. (b) The dry-based water content of CHI-100, CHI-90, and CHI-80 scaffolds. The values represent mean ± standard deviation, *n* = 4. ^∗^indicates *p* < 0.05 vs. PEG-100 and PEG-90; PEG-100, and PEG-80.

The compressive properties of the chitosan-PEG scaffolds were next evaluated (Figure 2(a)). The compressive stresses of all scaffolds increased with increasing strain until a maximum at the end of the compression (70% strain). The maximum compression stress of CHI-100, CHI-90, and CHI-80 scaffolds was 56.1 ± 2.0, 46.9 ± 1.6, and 41.3 ± 7.0 kPa, respectively. The incorporation of PEG significantly decreased the stiffness of chitosan scaffolds (*p* < 0.05). This situation is most visible in the CHI-80. It is not surprising because PEG is less stiffness material compared to chitosan [27]. A similar argument by Cheng et al. [28] explains the blend of PNIPAM with PEG hydrogels exhibits a lower mechanical strength than pure PNIPAM. Tanuma et al. [29] reported that the PEG-cross-linked chitosan hydrogel film swelling ratio increases with the decrease of molecular weight of PEG with the same content sample, and the degradation rate of chitosan component was found to be influenced by the content and molecular weight of PEG. An increase in the total PEG content resulted in a considerable increase in the degradation rate.

The water contents in CHI-100, CHI-90, and CHI-80 scaffolds were next determined. The water uptake of the chitosan scaffolds was significantly (*p* *<* 0.05) increased with increasing PEG contents from 4476 to 6025% (Figure 2(b)) dry weight basis. Besides the hydrophilicity of the added PEG, chitosan-PEG scaffolds have higher pore size and more water storage space as a result the ratio of water absorption had a significant difference (*p* *<* 0.05) with unmodified chitosan. The previous study on incorporating PEG into Alginate/Elastin composite matrix indicates water content increased with an increase in PEG content [30]. Similarly, Wan et al. [31] reported that the introduction of PEG segments enhanced the surface hydrophilicity of the poly-l-lactide-polyethylene glycol copolymers. Likewise, several modifications (chemical, mechanical, and structural) of hyaluronic acid hydrogels have been conducted in the fabrication of artificial extracellular matrix [32]. Since hyaluronic acid has negative charges, it can absorb large amounts of water and swell up to 1000 times in volume [33], However, chitosan is claimed for inadequate moisture availability, thus this study is the first on improving the hydrophilicity of chitosan scaffold via hydrophilic polymer along with the increase of pore size and water content.

Overall, the incorporation of PEG increases the pore sizes and the water-uptake ability of chitosan scaffolds but sacrifices the scaffold's stiffness. The Chitosan-PEG scaffolds with appropriate hydrophilicity were expected in favor of mass transportation, and then cell proliferation and differentiation. It was expected that cell proliferation would be much improved by increasing the hydrophilicity of the three-dimensional scaffolds, which even outweighed the disadvantages of the weaker mechanical property. Next, it was examined the effect of PEG incorporation in the culture of osteoblasts.

### 3.2. Osteoblast Culture on the Chitosan Scaffolds

**Figure 3:**
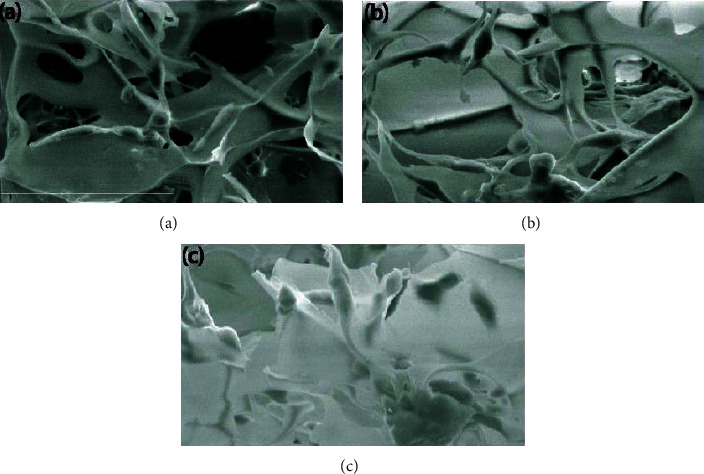
The morphology of the cells on (a) CHI-100, (b) CHI-90, and (c) CHI-80 scaffolds. The scale bar in the images represents 60 *μ*m.

**Figure 4:**
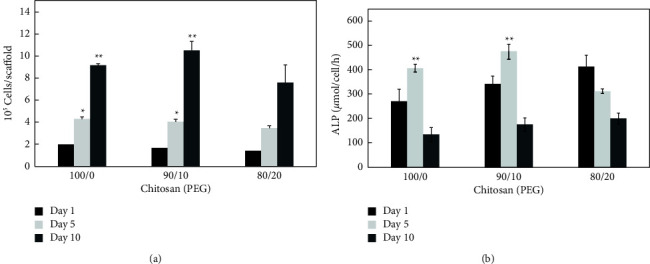
(a) Cell number of osteoblast after 1, 5, and 10 days of seeding on CHI-100, CHI-90, and CHI-80 scaffolds. The values represent mean ± standard deviation, *n* = 4. ^∗^indicates *p* < 0.05; ^∗∗^indicated *p* < 0.001 vs. Day 1 and Day 5. (b) ALP activity of osteoblast after 1, 5, and 10 days of seeding on CHI-100, CHI-90, and CHI-80 scaffolds. The values represent mean ± standard deviation, *n* = 4. ^∗^indicates *p* < 0.05; ^∗∗^indicated *p* < 0.001 vs. Day 1 and Day 5.

The nontoxicity of the Chitosan scaffold has been affirmed [34]. In this study, the chitosan-PEG scaffold showed good cell adhesion on all the used scaffold formulations (Figure 3). The cells on the pure chitosan scaffold (Figure 3(a)) are few and separately adhered on the surface, while the cells on chitosan-PEG (Figures 3(b) and 3(c)) are more aggregated which indicated the favored environment for cells proliferation. Cell proliferation is the process of multiplying the number of cells, and in this process, mitochondria gained a central role in the regulation of cell proliferation [35]. It was found that the addition of PEG decreased one-day cell adhesion to the chitosan-based scaffolds (Figure 4(a)). It is not surprising because PEG is a well-known nonadhesive material [36, 37]. After five days of incubation (Figure 4(a)), it was observed that the trend of cell number was still the same on the first day; however, the cell number in all scaffolds significantly (*p* < 0.05) improved and the doubling time of cells were 41.5, 22.6, and 23.3 h on CHI-100, CHI-90, and CHI-80 scaffolds, respectively. After 10 days of incubation, the cell numbers of CHI-90 (1.1 × 10^6^ cells/scaffold) surpass that of CHI-100 (9.2 × 10^5^ cells/scaffold), while the cell number of CHI-80 (7.6 × 10^5^ cells/scaffold) was significantly lower than the cell numbers of CHI-100 and CHI-90 (*p* < 0.001). During this period, the doubling time of cells of CHI-100, CHI-90, and CHI-80 scaffolds was 66.4, 21.5, and 926 h, respectively, indicating that the rate of cell proliferation of CHI-90 remained fast. However, the cell proliferation rate of CHI-100 and CHI-80 decreased, especially CHI-80.

PEG in chitosan scaffolds provides well hydration environment. As a result, it may enhance the diffusion of nutrients, bio-factors, and wastes. Hence, it might be the main reason CHI-90 scaffolds could maintain low doubling time. On the other hand, during incubation, it was observed that the CHI-80 scaffold was too soft that it might affect the cell proliferation of osteoblasts. It was reported before by Tanuma et al. [29] that the degradation rate of the chitosan component was found to be influenced by the content and molecular weight of PEG. An increase in total PEG content resulted in a considerable increase in the degradation rate.

The osteogenic differentiation of osteoblasts on the chitosan-PEG scaffolds was investigated by early and late osteogenic markers. Alkaline phosphatase (ALP), an essential enzyme for ossification, is an early bone marker protein, and one of the most frequently used markers to demonstrate osteoblast differentiation [38]. The final stage of osteoblast differentiation is mineralization, at which a mineral matrix containing mainly calcium phosphate is secreted and deposited by mature osteoblasts.

In this study, after osteogenic culture for one day, the cellular ALP activity of CHI-80 was the highest, followed by CHI-90 and CHI-100 (Figure 4(b)). However, after five days of incubation, the ALP activity of CHI-100 and CHI-90 increased significantly (*p* < 0.001) compared to their first day, respectively, and exceeded the values of CHI-80. After ten days of incubation, the ALP activity was decreased in all the samples. The ALP activity of CHI-90 was the highest on the fifth day, indicating the favored osteoblasts' early-stage differentiation.

**Figure 5:**
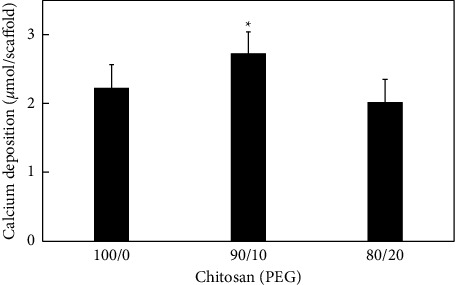
Calcium deposition of osteoblast following 15 days in osteogenic media in the CHI-100, CHI-90, and CHI-80 scaffolds. The values represent mean ± standard deviation, *n* = 4. ^∗^indicates *p* < 0.05; ^∗∗^indicated *p* < 0.001 vs. PEG-100 and PEG-90; PEG-100 and PEG-80.

After the osteoblasts were cultured in the osteogenic medium for 2 weeks, the total amounts of calcium in CHI-90 and CHI-100 were quantified as 2.7 and 2.2 *μ*mol/scaffold; whereas, the amount in CHI-80 was 1.9 *μ*mol/scaffold (Figure 5). The results indicate that calcium deposition between the CHI-100 and CHI-90 had a significant difference (*p* < 0.05), suggesting that osteoblast differentiation is enhanced with the optimal amount of PEG (10%). However, excess PEG (20%) significantly decreased osteoblasts mineralization. For future work, it is suggested to investigate optimizing hydrophilic polymer doping onto a chitosan scaffold.

## 4. Conclusion

The impact of PEG-incorporated chitosan scaffolds on osteoblasts differentiation and proliferation has been demonstrated in this study. The characteristic analysis of PEG-containing scaffolds exhibited bigger pore size, weaker mechanical properties, and higher water content compared to unmodified chitosan substrates. The cultured osteoblasts on the PEG-chitosan scaffold showed better cell proliferation and differentiation than that of the chitosan scaffold. However, adding more PEG (≥20% wt.) into the scaffolds has no benefit on the proliferation and differentiation of osteoblast. Taken together, these results indicate that adding hydrophilic molecules such as polyethylene glycol at an optimum amount (10% wt) into chitosan changed the characteristic of the scaffolds and improved the proliferation and differentiation of osteoblast. The biocompatibility, safety, and biodegradability of the chitosan make it an excellent scaffold candidate, and in the near future will witness its crucial role in biomaterials and tissue engineering.

## Figures and Tables

**Table 1:** The weight percentages of the compositions in the scaffolds.

Type of scaffolds	Chitosan	CHI-g-AZ	CHI-g-RGD	PEG
CHI-100	40	50	10	0
CHI-90	30	50	10	10
CHI-80	20	50	10	20

## Data Availability

All data used to support the findings of this study are included within the article.
